# Bronchodilator-responsive bronchiolar obstruction in term neonates: a case series 

**DOI:** 10.1186/s13256-023-04035-4

**Published:** 2023-07-31

**Authors:** Beatrice N. Ezenwa, Abdou Gai, Ellen Kujabi, Abdoulie Garba, Yarreh Suso, Abdulwahab Sallah, Egbuna O. Obidike

**Affiliations:** Department of Paediatrics, Edward Francis Small Teaching Hospital (EFSTH), Banjul, Gambia

**Keywords:** Bronchiolar obstruction, Term neonates, Wheezing, Bronchodilators, Reactive airway, Airway hyperresponsiveness

## Abstract

**Background:**

Bronchiolar obstruction, which causes airway obstruction in hyperresponsive airways, often results from the contraction of the airway's smooth muscles, increased viscid mucous secretions, and mucosal oedema consequent upon a reduced cyclic 3,5-adenosine monophosphate (c-AMP). These processes respond to bronchodilators. The six cases presented to us, in Edward Francis Small Teaching Hospital (EFSTH), Banjul, The Gambia, in the newborn period with clinical features suggesting obstruction with airway reactivity with response to bronchodilator treatment are presented here. Our capacity-limited literature search did not show any such report in neonates. This report highlights the need for this condition to be sought in neonates, medically managed in resource-poor countries without resorting to high-cost equipment use, and for its possible future classification.

**Case presentation:**

We report six cases of Gambian neonates consisting of four males and two females ages 2–27 days who presented to us with histories of fast breathing of a few hours duration and expiratory respiratory distress. All were term babies with rhonchi and demonstrable prolonged expiration with terminal effort. They all had a diagnosis of hyperreactive airway disease with bronchiolar obstruction. Five cases were first-time wheezers, while one was a recurrence. All were eventually treated with bronchodilators and steroids with good results. The median duration for resolution of most symptoms with treatment was two days, with a range of 1–5 days.

**Conclusion:**

Clinically determined bronchiolar obstructions in term neonates can be relieved with bronchodilators and steroids, and this treatment modality, if employed where the pathological process can be established, can reduce the demand on scarce resources in resource-poor countries.

## Background

Obstructive airway disorders in neonates are a common and potentially serious condition. This can occur in the lower airway because they are very collapsible and made of cartilaginous and loose connective tissues [1]. When there is an additional narrowing of this airway caused by inflammatory and mucosal edema, tenacious mucosal secretions, and bronchoconstriction of the airway's smooth muscles [2], a bronchiolar obstruction results—a lower airway obstruction. Bronchoconstriction, a state of bronchial smooth muscle tone is modulated by the interactions of the beta-adrenergic receptors and the metabolism of the intracellular cyclic nucleotides. Beta-adrenergic stimulation increases cyclic 3,5-adenosine monophosphate (AMP) and decreases cyclic 3,5-guanosine monophosphate (GMP) [2]. The principal process by which intracellular cAMP levels are reduced is via hydrolysis by the phosphodiesterase enzymes. Phosphodiesterase inhibitors, such as the xanthines, by inhibiting this enzyme increase the cytosolic cAMP [3].

Cyclic AMP (cAMP) plays a crucial role in neonatal airway function, tone, and caliber. It controls the airway smooth muscle (ASM) tone by inhibiting the contraction of the ASM [3]. The increased cAMP acting on submucosal glands influences water fluxes and hydration status across the airway epithelium with a reduction in the viscosity and tenacity of the secreted mucus [4, 5]. Cyclic AMP also controls the clearance of the airway mucus by modulating the ciliary beat frequency [6] and suppresses the pro-inflammatory activities of the body's immune and inflammatory cells. However, its role in ASM relaxation in newborns is not optimal as the newborn ASM is underdeveloped at birth. The ASM continues to develop in early infancy.

Bronchiolar obstruction in reactive airway is typically seen in children with asthma, bronchiolitis, and airway hyperresponsiveness, causing rapid airway narrowing, which are usually reversible [7]. Most respiratory disorders that cause airway narrowing or obstruction can cause wheezing. Heightened airway reactivity has been proposed as one possible explanation for the increased occurrence of wheezing in infants [8].

A number of risk factors have been identified to increase the occurrence of reactive airway diseases or bronchiolar obstruction [9]. These include not being exclusively breastfed, viral respiratory infections like RSV, genetic predisposition, and allergic responses to environmental triggers.

Bronchiolar obstruction in children is characterized by wheezing, hyperinflation of the chest, and signs of respiratory distress [10]. Other recognized features of the diseases in the neonatal age include noisy and difficult breathing, fast breathing, fever, cough, and wheezing. The wheeze is caused by increased airway resistance, narrowing, and closure of the small airways, which are more marked during expiration [11]. In bronchiolitis, wheezing during inspiration and expiration suggests a more severe airway narrowing [2]. Affected babies may be diagnosed by history and physical examinations during early infancy and by spirometry in older children.

The use of normal saline nebulization to decongest, soothe, moisturize, and deliver inhaled medications has been reported for many respiratory disorders [12]. Pressure support is another form of treatment that can be offered in airway hyperresponsiveness. According to a Cochrane Database Systematic Review, pressure support of the airways with CPAP can prevent the collapse of poorly supported peripheral small airways during expiration [13].

Beta adrenoceptor agonists (such as nebulized salbutamol) and phosphodiesterase inhibitors such as intravenous aminophylline have been used effectively in treating bronchiolar obstructions in early infancy with conflicting results [14–16]. Many researchers have opined that bronchodilators do not affect ASM tone in early infancy. Therefore, using bronchodilators in bronchiolitis and reactive airway diseases in early infancy was discouraged. This argument focuses on a single mechanism of action of the bronchodilators and would seem to discountenance the other possible effects of these medicines. Bronchodilators such as B2 agonists through cAMP-mediated pathways and phosphodiesterase inhibitors through their actions function by increasing the cytosolic cAMP levels, which then modulates the cellular events as had been stated earlier [3].

To enhance the action of the beta-agonists on the ASM, steroids are also used in airway reactivity [17, 18]. At the cellular level, steroids reduce the recruitment, numbers, and survival of inflammatory cells in the hyperresponsive airways and suppress the production of chemotactic mediators in the epithelial cells of the airways [17]. They reverse mucosal edema, decrease vascular permeability by vasoconstriction and reduce mucus secretions in the airways [18].

We report six cases of neonates that presented in our facility with features of bronchiolar obstruction and were treated with salbutamol and /or aminophylline and steroids with good results. We highlight significant findings that may help diagnosis in neonates.

## Case presentation

We reviewed the case notes of the six presentations of five Gambian neonates who were brought to our neonatal emergency with difficult and noisy breathing of variable durations before the presentation between September 2022 and January 2023. The baseline characteristics are shown in Table 1. All were term babies. Only two babies, BN and LB had a family history of asthma. MJ had associated skin eruptions diagnosed as impetigo at presentation. LB had Down Syndrome features and a second admission with similar wheezy symptoms within two weeks after the first discharge.

**Table 1:** Baseline characteristics of the patients

Serial number	Patient identification	Ethnicity	sex	Age at admission (days)	Gestational age	Weight at presentation (g)	Referred from
1	B.N	Mandinka	M	21	Term	3200	Home
2	M.S	Fula	F	2	39 weeks	2320	EFSTH maternity ward
3	M.C	Mandinka	F	15	Term	3500	BDH
4	M.J	Jola	M	5	Term	3000	BDH
5	L.B_1_	Wolof	M	18	Term	2525	BDH
6	L.B_2_	Wolof	M	27	Term	2855	Home

*EFSTH* Edward Francis Small Teaching Hospital, *BDH* Brikama District Hospital

Table 2 shows the major clinical findings at admission. All the babies had rhonchi and demonstrable prolonged expiration with terminal effort. All were treated with oxygen and nebulized with salbutamol and intravenous corticosteroid with good results. Intravenous aminophylline was also administered to M.J. after three days on salbutamol with little improvement. He was also treated for impetigo with antibiotics. L.B. was treated with nebulized isotonic saline on his first admission, but on his second admission, he was treated with salbutamol and hydrocortisone. The median duration for resolution of tachypnoea for the cohort was two days, with a range of 1–5 days while the median duration of hospital stay was five days, with a range of 3–10 days. All the babies had been seen at least once in the follow-up clinics and doing well.

**Table 2:** Clinical features at admission, treatment, and outcome

Characteristics	B.N	M.S	M.C	M.J	L.B_1_^**^	L.B_2_^**^
Coryza	Present	Absent	Present	Present	Absent	Absent
Fever	Absent	Absent	Absent	Absent	Absent	Absent
Difficult breathing	Present	Present	Present	Present	Present	Present
Duration of symptoms before presentation	5 h	1 h	8 h	4 h	72 h	24 h
Feeding type	Human milk	Formula milk	Human milk	Human milk	Mixed feeding	Mixed feeding
Use of accessory muscles of respiration	Present	Present	Present	Present	Present	Present
Tachypnoea	76	79	72	83	72	62
SPO_2_ (%) in room air	98	72	78	91	95	94
Prolonged expiration	Present	Present	Present	Present	Present	Present
Expiration with terminal effort	Present	Present	Present	Present	Present	Present
Percussion note	Not done	Hyper-resonant	Hyper-resonant	Hyper-resonant	Hyper-resonant	Hyper-resonant
Rhonchi	Present	Present	Present	Present	Present	Present
FBC report	Not suggestive of sepsis	Not suggestive of sepsis	Not suggestive of sepsis	Not suggestive of sepsis	Not suggestive of sepsis	Not suggestive of sepsis
CXR report	Not done	Features of hyper-inflation	Not done	Not done	Not done	Features of hyper-inflation
Treatment given	Intranasal oxygen, nebulized with Salbutamol and Dexamethasone	Intranasal oxygen, nebulized with Salbutamol and intr avenousHydrocortisone	Intranasal oxygen, nebulized with Salbutamol and intr avenousHydrocortisone	Intranasal oxygen, nebulized with Salbutamol, intravenous Aminophylline and Hydrocortisone	Nebulized with Normal saline	Nebulized with Salbutamol and Hydrocortisone
Time to resolution of tachypnoea (Days)	2	2	4	5	2	1
Duration of hospital stay (Days)	5	3	8	10	3	4
Outcome	Discharged	Discharged	Absconded	Discharged	Discharged	Discharged

**Same child was admitted twice with similar symptoms

*FBC* Full blood count,* CXR* chest X-ray

## Discussion

Airway reactivity with wheezing in neonates presents a diagnostic dilemma, and the tendency to miss the diagnosis is high. This is majorly about what to call it and perhaps this has resulted in it not being looked for in the neonate. The six, term neonates with a male: female ratio of 3:2 in this series presented between 2 and 21 days of life in the first instance with features of bronchiolar obstruction or reactive airway diseases. Information regarding this condition in term neonates was not found in the literature searched.

Lack of breastfeeding and feeding with formula milk has been documented as risk factors for reactive airway diseases in children [19, 20]. Three of our cases were on formula feeds. The mother was supplementing human milk with formula feeds in the child who presented twice. Breast milk is laden with immunogens that protect infants from allergies by suppressing immune reactions that can trigger wheezing [21]. From the 50% of cases from our series who were exclusively on human milk feeds, this protection may not have been activated or their reactivity is from things this protection cannot be effective against—one of these babies was among those with a family history of asthma while the two others did not. Two of the cases we reported had a family history of asthma while one child had a recurrence. Unlike in the newborn, a history of recurrent wheezing episodes and a family or personal history of asthma, nasal allergies, or eczema will help to support a diagnosis of asthma [22]. In the newborn, a precise diagnosis for this condition is not often made and a search through the literature was not helpful.

Though three of the six cases in this series had coryza, this may not say much. All the patients showed manifestations of respiratory distress with severity on expiration and a hyper-resonant chest on percussion. These are generally in keeping with findings in bronchiolar obstruction [21]. The two patients with a chest x-ray showed features supporting bronchiolar obstruction.

The six neonates presented here showed evidence of respiratory distress on expiration with rhonchi (wheeze) suggesting bronchiolar obstruction. Wheezing in the neonatal age is usually first-time wheezes. Researchers have documented that many first-time wheezes are caused by viruses, of which RSV has been a chief culprit [23]. This may not be plausible in the neonatal age as most babies are protected by maternal antibodies to these viruses early in life [24]. This puts a question mark on the cause of the symptoms in our patients. This question is reinforced by how early these symptoms started from birth. Though we did not try to document etiology, our findings tend to suggest some element of familial predisposition or reaction to some noxious agents but not necessarily infective. This is because the short interval of symptoms of less than 24 h before presentation in most of the patients would suggest more of a reaction than an infective process. This thinking, if true, may confer an advantage because it has been documented that infection during the neonatal age can adversely affect the developing lung and immune system. Al-Garawi* et al*. in an animal model, demonstrated that infection during the neonatal period can increase airway hyperresponsiveness in mice which may persist to adulthood [25]. Notwithstanding this possible advantage if not due to an infection, it has however been documented that increased airway reactivity in term neonates was also associated with an increased risk of developing airway disease and lower lung function later in life [8].

None of our patients had fever at presentation, thus collaborating with the complete blood count result findings which were not suggestive of sepsis, even though a CRP which could not be done may have been a better indicator.

All the patients responded to nebulization with various agents prominent among which was salbutamol. One patient had an additional introduction of intravenous aminophylline after three days of poor response. Five of the six cases also received steroids on admission. The case that did not receive steroids was nebulized with normal saline. He relapsed within two weeks and had to be treated with salbutamol and steroid at readmission.

Ordinarily, bronchodilators are thought to act at the level of the ASM. For newborns, ASM is immature. This raises a question as to why our patients responded to the bronchodilators. In airway reactivity, there are three events occurring, and considering that the airway lumen in the newborn is narrow, any of the triple events of bronchial hyperresponsiveness viz bronchoconstriction, increased secretion, and viscosity of the secretions, and mucosal edema will be capable of creating some degree of obstruction. As these neonates had clinically determined bronchiolar obstruction that responded to bronchodilators and steroids, it is very likely that the other two pathways, apart from the ASM contraction, were at play causing the obstruction. These two pathways are known to equally respond to the use of cAMP modulators [3, 6] as indicated by the resolution of symptoms in our patients. Another treatment modality that is said to be effective will include the use of CPAP which has been shown to keep the airway patent [13] and this could have sometimes been used for this condition in situations where there was bronchiolar obstruction ab initio but was not determined. Humidifying the airway by nebulizing with isotonic saline to reduce the viscosity of the tenacious mucus is also a treatment modality [12]. The use of normal saline nebulization to decongest, soothe, moisturize, and deliver inhaled medications has been reported for many respiratory disorders. Barski* et al*. reported that isotonic saline reduces breathlessness and provides respiratory relief when used alone or in combination with other therapies [12]. While the systematic analysis by House* et al*. recommended nebulized isotonic saline as a treatment for airway hyperresponsiveness such as in bronchiolitis [26], our experience suggests differently as our patient that used it, though he improved, he relapsed within two weeks and had to be re-admitted and then treated with a beta-agonist and steroids.

From the findings in this study, it is obvious that a careful and proper clinical assessment of a patient will give a clue as to the pathological process going on and this will likely determine the treatment modality to be employed.

As has been reported by some workers, there is a need to fashion a better name for this "reactive airway disease" in all ages [27], especially in neonates whose wheeze does not fit into any of the regular definitions in use as accurate diagnosis and treatment will reduce unnecessary and prolonged hospital stays and also reduce pressure on the scarce facilities available in low-resource settings. The neonates that present with lower airway obstruction features—airway edema, inflammation, and mucus secretion—and respond to treatment with the beta-agonists, xanthines, and corticosteroids will need to be appropriately classified.

## Conclusion

The six cases in this study have indicated that clinically determined bronchiolar obstructions in term neonates can be relieved with bronchodilators and steroids and this treatment modality if employed where the pathological process can be established can reduce the demand on equipment, especially in resource-poor countries.

Nebulized isotonic saline may not proffer lasting relief in neonatal airway hyperresponsiveness.

There is a need for further multicenter studies to examine this condition and follow up on these patients and similar cases to assess their long-term outcomes.

## Data Availability

Not applicable.

## Abbreviations

ASM: Airway smooth muscle
cAMP: Cyclic 3,5-adenosine monophosphate
GMP: Cyclic 3,5-guanosine monophosphate
CPAP: Continuous positive airway pressure
CRP: C-reactive proteins
RSV: Respiratory syncytial virus
